# A distribution-wide dataset of Atlantic walrus terrestrial haul-out sites

**DOI:** 10.1038/s41597-026-07210-6

**Published:** 2026-04-09

**Authors:** Hannah C. Cubaynes, Cory J. D. Matthews, Eva Garde, Maria Gavrillo, Mads Peter Heide-Jørgensen, Jeff W. Higdon, Kit M. Kovacs, Margarita Leskova, Christian Lydersen, Boris Solovyev, Alejandra Vergara-Pena, Peter T. Fretwell

**Affiliations:** 1 British Antarctic Survey, Cambridge, United Kingdom; 2 Fisheries and Oceans Canada, Winnipeg, Canada; 3 Greenland Institute of Natural Resources, Nuuk, Greenland; 4 Arctic and Antarctic Research Institute, St Petersburg, Russia; 5 Higdon Wildlife Consulting, Manitoba, Canada; 6 Norwegian Polar Institute, Tromsø, Norway; 7 Systematic Conservation Consultancy, Sydney, Australia; 8 WWF-UK, Woking, United Kingdom

**Keywords:** Conservation biology, Marine biology

## Abstract

The Atlantic walrus (*Odobenus rosmarus rosmarus*) is an Arctic endemic species that is under increasing threat due to declines in their sea ice habitats. Walruses rely directly (*e.g*., resting, giving birth) and indirectly (*e.g*., tight coupling between sympagic and benthic productivity) on sea ice, and are particularly sensitive to disturbance by increasing anthropogenic activities (*e.g*., shipping) that are taking place concomitant with sea ice declines throughout their Arctic range. Management and conservation of Atlantic walrus require monitoring of their distribution and assessments of regional and range-wide abundance trends. Atlantic walrus population assessments are typically based on counts of walruses hauled out at terrestrial sites during aerial or boat-based surveys, and increasingly, using satellite imagery. We compiled a comprehensive, distribution-wide dataset of all known Atlantic walrus terrestrial haul-out sites from relevant national datasets to promote accessibility and consistency of use among multiple users, including Indigenous groups, scientific researchers, and resource managers.

## Background & Summary

**Fig. 1:**
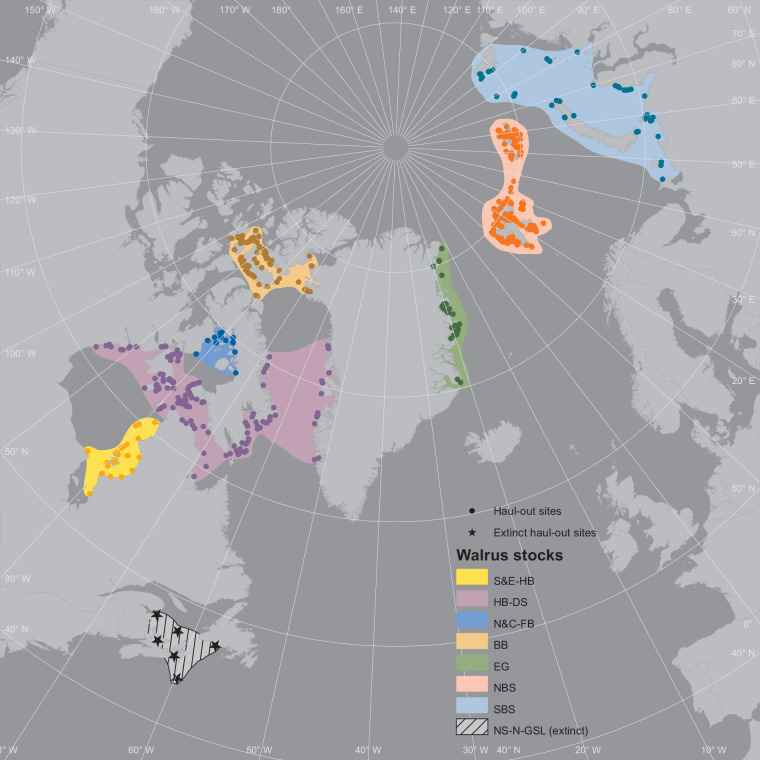
Map of the Atlantic walrus (*Odobenus rosmarus rosmarus*) management stocks and all the terrestrial haul-out sites listed in the dataset.

Walruses (*Odobenus rosmarus*) are an Arctic-endemic species with a near circumpolar range. The Atlantic subspecies *O. r. rosmarus* is distributed from the Canadian Arctic eastward to the Kara Sea (Fig. 1). The remaining regions of the Arctic are occupied by the Pacific subspecies (*O. r. divergens*). Throughout their range, walruses are facing growing threats due to global warming inducing declines in sea ice, on which they depend for resting and giving birth^1–3^. Walruses forage almost exclusively on benthic organisms, and changes in the tight coupling between ice algae production’s delivery of nutrients to benthic communities is likely to impact food availability for walruses^4–6^. Increased shipping with declining sea ice has also been identified as one of the key anthropogenic threats to walruses^7^. Walruses are sensitive to shipping disturbance^8,9^, which can cause stampedes. These have been reported to lead to injuries and death^2,7,10–12^, and abortion of foetuses^2^, as well as long-term abandonment of haul-out sites (and associated preferred foraging sites)^2^. Reduced sea ice might also lead to increased predation (*e.g*., from killer whales, *Orcinus orca*, and polar bears, *Ursus maritimus*, as their seasonal and geographical overlap with walruses is expected to increase) and exposure to pathogens (*e.g*., temperate-adapted species migrating to the Arctic and carrying disease walruses have not previously been exposed to)^13^.

Walruses are a sentinel species that can signal environmental changes taking place in their communities. The Conservation of Arctic Flora and Fauna (CAFF) recognises walruses as a focal ecosystem component (FEC) due to the key role they play in the Arctic ecosystem^14^ and their cultural and economic importance to Arctic residents^15^. The International Union for Conservation of Nature (IUCN) classified walruses as vulnerable on the global Red List because walrus populations are predicted to decline due to the loss of sea ice^16^. Therefore, systematic monitoring of walrus populations, particularly abundance trends and changes in distribution, is required to assess how walruses are responding to loss of sea ice habitats and anthropogenic disturbances. Walruses are also hunted in several areas of the Arctic and monitoring population trends is critical for the assessment of the sustainability of the hunt. Atlantic walrus population assessments are often based on counts of walruses at terrestrial haul-out sites in the summer months when sea ice is at its annual minimum, and when the maximum numbers of walruses are hauled out on land to rest between foraging trips^2,17^. Walruses exhibit strong interannual site fidelity to certain terrestrial sites over long periods of time that are typically surveyed using aircraft and boats, and increasingly, satellite imagery^17–25^. For Pacific walruses (*O. r. divergens*), all known terrestrial haul-out sites are published in one dataset^20^. However, locations and other relevant metadata for Atlantic walrus haul-out sites are spread among many different datasets and publications. To promote accessibility of haul-out site data, as well as consistent use across multiple users and jurisdictions, we have compiled a comprehensive dataset of known Atlantic walrus terrestrial haul-out sites from various disparate sources, which we have made available on the Arctic Biodiversity Data Service of CAFF. This dataset will be kept up to date.

## Methods

### Compiling the dataset

We compiled the dataset^26^ by merging existing datasets^27,28^ and incorporating all available information from published scientific and Indigenous works. The merged dataset is largely a combination of national and other jurisdictional datasets maintained for the purpose of regular walrus stock assessments. These, in turn, have been compiled through consultations with indigenous Knowledge Holders (*e.g., Inuit Qauijimajatuqangit*, or IQ), historical records, and observations during dedicated coastal surveys of walrus abundance and distribution (see the user’s guide document included with the dataset for a detailed list of all references used to compile the dataset).

### Dataset attributes

This dataset includes all sites where walruses have been observed on land. Although each haul-out site is identified by a coordinate, location certainty ranges from high (*e.g*., records with GPS measurements during surveys) to uncertain for records from some sources (*e.g*., historic reports). Each haul-out site record is characterised by the same set of information, which is described in Table 1.

**Table 1:** Description of each field for the Atlantic walrus terrestrial haul-out site dataset.

Field	Description
ID_CAFF	Identification number unique to each haul-out site for the dataset uploaded on the Arctic Council Conservation Arctic Fauna and Flora.
HAUL_NAME	Haul-out site name.
ALT_NAME	Alternative haul-out site name(s).
LATITUDE	Latitude in decimal degree (WGS 1984).
LONGITUDE	Longitude in decimal degree (WGS 1984).
AREA	Area of one or more stocks as defined by NAMMCO^29^. See Table 2 and Fig. 1.
STOCK	Walrus stock as defined by NAMMCO^29^. See Table 2 and Fig. 1.
COUNTRY	Country where the haul-out site is located.
SPATIAL_AC	Spatial accuracy (high, moderate, uncertain) as defined by Higdon (2016)^28^. **High:** exact coordinate provided. **Moderate:** position estimated from map(s) with sufficient resolution to have reasonable certainty of accuracy within 5 km. **Uncertain:** position estimated from map(s) with insufficient resolution (or landscape pattern adds uncertainty), from non-spatial sources, or for haul-out sites where different sources locate them differently or show different numbers of sites in particular locations. Certainty of accuracy is greater than 5 km.
STATUS	Status of the haul-out site (active, not in use, uncertain, extinct) as defined by Higdon (2016)^28^ with the added class “extinct”. **Active:** currently used by walruses (*i.e*., published evidence of recent use in the last 10 years or reported to be active by local communities). **Uncertain:** no recent data to confirm or suggest current use. **Not in use:** haul-out site previously reported to be abandoned, with no recent data to refute this. **Extinct**: haul-out site from population reported to be extinct.
LAST_SEEN	The year walruses were last observed at a given haul-out site.
LARGEST_N	Largest number of walruses recorded at the haul-out site.
LGST_N_10Y	Largest number recorded in approximately the last 10 years at the haul-out site.
LAST_SRVD	Year the haul-out site was last surveyed.
LST_SRV_BY	Who last surveyed the haul-out site.
TIME_PRES	Time when walruses are known to be present at the haul-out site.
PRIM_SOURC	Primary source.
OTR_SOURCS	Other sources.
COMMENTS	Any notes thought to be helpful.

Each haul-out site in the dataset was assigned to a stock recognised by NAMMCO^29^, with two exceptions (Table 2 and Fig. 1) which are:

1. Northern Foxe Basin and Central Foxe Basin are joined together as the North and Central Foxe Basin Stock (N&C-FB) because no geographical distinction can be made between animals at these sites: walruses from these stocks can only be differentiated based on their morphology and isotopic differences^30–32^.
2. The Nova Scotia-Newfoundland-Gulf of St Lawrence (NS-N-GSL) stock is not included in NAMMCO, because it is extinct^2,33–35^. However, we include it herein to ensure comprehensiveness, given that we include all historical and active haul-out sites.

**Table 2:** List of management stocks and their corresponding areas of occupancy for Atlantic walruses based on definitions and terminology of NAMMCO^29^. See also Fig. 1.

Stock	Area
Baffin Bay (BB)	High Arctic
North and Central Foxe Basin (N&C-FB)	Central Arctic
Hudson Bay – Davis Strait (HB-DS)	
Southern & Eastern Hudson Bay (S&E-HB)	
Nova Scotia-Newfoundland-Gulf of St Lawrence (NS-N-GSL) (***Extinct***)	Nova Scotia-Newfoundland-Gulf of St Lawrence
East Greenland (EG)	Greenland Sea
Northern Barents Sea (NBS)	Barents Sea
Southern Barents Sea (SBS)	

### Long-term dataset management

This Data Descriptor describes a static dataset that was peer reviewed at the time of article submission and acceptance. Future updates will be reviewed and the dataset updated as needed by members of CAFF’s Marine Mammal Expert Group, who will remain in contact with national walrus experts and researchers surveying Atlantic walruses. These will be assigned a different identifier and may be found via the repository page.

## Data Records

The dataset (identifier: fcdc5e58-3318-4a02-a484-8ecb7cfb66e2) is available on the Arctic Biodiversity Data Service of CAFF^26^. It contains the dataset available either in a Comma Separated Values (CSV) file or a shapefile (SHP) format and a user’s guide. The dataset includes 499 terrestrial haul-out sites, spread across Canada (237 sites), Greenland (51 sites), Norway (125 sites) and Russia (86 sites).

## Technical Validation

We mapped all haul-out sites using a geographic information system software suite (ESRI ArcGIS Desktop 10.8.0.12790) and ESRI World Imagery basemap (ID: 10df2279f9684e4a9f6a7f08febac2a9). Local experts ensured locations were correctly placed before exporting the dataset as a CSV file.

## Usage Notes

The geographic coordinate system for all coordinates is WGS 1984 (EPSG: 4326). For best visualisation, we recommend projecting the dataset in North Pole Stereographic (EPSG: 102018) or North Pole Azimuthal Equidistant (EPSG: 102016).

### Potential applications

This dataset has applicability for any walrus monitoring effort aiming to assess population trends (*i.e*., abundance estimates) and distribution/range that rely on surveying walruses at their terrestrial haul-out sites. Applications extend to the use of emerging tools, such as tasking locations for satellite imagery collection^25^. This is particularly relevant if using very high-resolution satellite imagery because these types of satellites do not generally capture imagery unless an order is placed. The Walrus from Space project (https://www.wwf.org.uk/learn/walrus-from-space)^36^, which aims to monitor Atlantic walrus populations across their range, has been making use of this dataset. Applications also extend to marine planning, such as regulation of shipping routes, or in case of oil spills.

## Data Availability

The dataset^26^ is available on the Arctic Biodiversity Data Service of CAFF, accessible at the following link: https://geo.abds.is/geonetwork/srv/eng/catalog.search#/metadata/fcdc5e58-3318-4a02-a484-8ecb7cfb66e2.

## Code availability

The dataset was compiled manually from existing national or other jurisdictional datasets and complemented with published work mentioning terrestrial haul-out sites, without the use of coding.
